# Quality of life, depression, anxiety and suicidal ideation among men who inject drugs in Delhi, India

**DOI:** 10.1186/1471-244X-13-151

**Published:** 2013-05-27

**Authors:** Gregory Armstrong, Amenla Nuken, Luke Samson, Shalini Singh, Anthony F Jorm, Michelle Kermode

**Affiliations:** 1 Nossal Institute for Global Health, University of Melbourne, Victoria, Australia; 2 The Society for Service to Urban Poverty (SHARAN), Delhi, India; 3 Population Mental Health Group, Melbourne School of Population Health, University of Melbourne, Victoria, Australia

## Abstract

**Background:**

Mental disorders such as depression, anxiety and suicide represent an important public health problem in India. Elsewhere in the world a high prevalence of symptoms of common mental disorders have been found among people who inject drugs (PWID). Research in India has largely overlooked symptoms of common mental disorders among this high risk group. This paper reports on the results of a survey examining quality of life, depression, anxiety and suicidal ideation among adult males who inject drugs living in Delhi.

**Methods:**

Participants (n = 420) were recruited from needle and syringe programs using time location sampling and were interviewed using an interviewer-administered questionnaire. Self-report symptom scales were used to measure the severity of symptoms of depression (PHQ-9) and anxiety (GAD-2) within the preceding 2 weeks. We assessed the presence of suicidal thoughts and attempts within the past 12 months.

**Results:**

The mean length of injecting career was 20.9 years indicating a sample of chronic injecting drug users, of whom only one-third (38%) were born in Delhi. The level of illiteracy was very high (62%), and just 2% had completed class 12. Scavenging / rag picking was the main form of income for 48%, and many were homeless (69%). One-third (33%) had been beaten up at least twice during the preceding 6 months, and many either never (45%) or rarely (27%) attended family events. We found a high prevalence of depressive (84%, cut-off ≥10) and anxiety (71%, cut-off score of ≥3) symptoms. Fifty-three percent thought about killing themselves in the past 12 months, and 36% had attempted to kill themselves.

**Conclusions:**

Our findings revealed a socially excluded population of PWID in Delhi who have minimal education and are often homeless, leaving them vulnerable to physical violence, poverty, poor health, imprisonment and disconnection from family. The high prevalence of psychological distress found in this study has implications for programmes seeking to engage, treat and rehabilitate PWID in India.

## Background

Mental disorders are one of the leading contributors to the global burden of disease in both high and low income countries [1]. Common mental disorders such as depression and anxiety are frequently comorbid with other health and psychosocial problems and are among the most important causes of morbidity [1-3]. Mental health remains a low priority in most low and middle income countries and unmet needs for treatment are pervasive [4-6].

Mental disorders constitute a major public health problem in India [7-9]. Epidemiological studies have reported prevalence rates for psychiatric disorders varying from 9.5 to 370/1000 population, with prevalence highest among marginalised populations and those most disadvantaged by the phenomenon of rapid economic growth and urbanization, such as urban slum dwellers [10]. High rates of suicide have been documented in India [11-14]. The real frequency of attempted suicide is thought to be under-reported due to a poor reporting system and the social taboos and legal ramifications (attempted suicide is a punishable offence under Section 309 of the Indian Penal Code) connected to suicide [12,15].

Previous research has documented the co-occurrence of mental disorders and illicit substance use [16,17]. The association can be explained in different ways. Psychiatric disturbance can occur before or after the commencement of illicit substance use: people may use illicit substances to manage their psychiatric symptoms; the pharmacological properties of illicit substances such as amphetamines can induce psychiatric symptoms; the symptoms of withdrawal connected with drug addiction can mimic those of common mental disorders; and, the psychosocial stressors connected with life for people who use illicit substances create distress and can have a detrimental effect on mental health [18]. The precise origin of psychiatric symptoms may not be so critical given that comorbid psychiatric symptoms in substance users are connected with a poor long-term prognosis regardless of whether these symptoms were independent of the substance use or were a consequence of it [19-21].

A high prevalence of symptoms of depression, anxiety and suicidal ideation has been found among people who inject drugs (PWID) in high-income country settings using different diagnostic methods, instruments, cut-off values, and population samples [22-26]. Few studies have examined the phenomenon of symptoms of common mental disorders among PWID in Asian country settings, and those few studies that have been undertaken have found similarly high levels of psychiatric symptomatology [27-30]. To the best of our knowledge, there has been only one community-based study among PWID in India that measured the prevalence of suicidal ideation and that was in the context of a study focused on human rights violations [31]; there have been no studies using community-based samples that measured depression or anxiety symptoms.

This paper reports on the results of a cross-sectional survey examining quality of life, depression and anxiety symptoms, and suicidal ideation among a community-based sample of adult males who inject drugs living in Delhi. The populations of PWID in Delhi, estimated to be at around 35,000 [32] are from various walks of life, but most are substantially alienated and impoverished and live at the margins of society [33]. Our study of symptoms of common mental health problems among PWID in Delhi will have implications for public health programmes targeting this population.

The research questions framing our study were: (1) what are the quality of life and socio-economic characteristics of this population of PWID?; (2) what is the prevalence of symptoms of depression and anxiety and suicidal ideation?; (3) what psychosocial factors are associated with symptoms of depression, anxiety and suicidal ideation?; and, (4) is there an association between risky injecting and sexual practices and symptoms of depression, anxiety and suicidal ideation? This paper will only report on the first and second research questions to allow for a full discussion of the characteristics of this population. The other objectives will be reported in depth elsewhere.

## Methods

### Study design

In April and May of 2012, a cross-sectional survey was undertaken among PWID in Delhi using a structured questionnaire that was interviewer-administered. Ethics approval was provided in Australia by the Human Research Ethics Committee at The University of Melbourne (HREC 1137025). Local ethics approval was provided in India by the Institutional Review Board (IRB) at Sharan, The Society for Service to Urban Poverty. Sharan is an NGO that provides services to injecting drug users in New Delhi and is the partner NGO on this study.

### Participants

Eligible participants were those who were 18 years of age or older, had injected drugs at least once in the past month, were not currently enrolled in opioid substitution therapy (i.e. buprenorphine), and had given their informed oral consent to participate in the study. A consent form and plain language statement were orally communicated to potential participants, as literacy was limited for many participants. Consent was obtained orally after reading out the consent form. Participation in the study was voluntary and anonymous – no names or individually identifying information were recorded at any stage. Participants were provided with a small meal (e.g. chai and samosa) for their time in participating in the study. Participants were free to withdraw from the study during the questionnaire without losing access to the meal.

Participants were sampled from three needle and syringe programmes (NSPs) in Delhi, each located in a distinctly different geographical area so that the sample was drawn from a diverse range of PWID from across Delhi; 1) Yamuna Bazar, 2) Nabi Karim, and 3) Jahangirpuri. Yamuna Bazar is located on the bank of the Yamuna River in central Delhi and is the base for a large proportion of the capital’s homeless and PWID. Jahangirpuri is a slum resettlement colony on the outskirts of metropolitan Delhi. The NSP at Nabi Karim is located in an urban ghetto in the Muslim quarter adjacent to New Delhi Railway Station.

According to current data presented in the recent National AIDS Control Organisation (NACO) Annual Report 2011–2012 [34], it is estimated that 80% of the officially mapped IDU population usually obtain their needles from NSP targeted interventions depending on the location.

### Sampling method

The data were collected using Time Location Sampling (TLS), a recognized method for obtaining a probability-based sample from hidden populations [35-38]. TLS sampling involves building a sampling frame from a list of location and time combinations based upon the locations and times where you would expect to find members of the target population. The NSPs were mapped in terms of their operating hours and a list was constructed based on a combination of the locations (i.e. NSPs) and blocks of time (e.g. 3 hour blocks) during which the NSP would be operating. Combinations of location and time, known as sampling events, were then randomly selected from the list to populate a sampling calendar. The random number generator in Excel was used to randomly select sampling events for each date on the sampling calendar. The sampling calendar detailed the location and time that the data collection team would attend on a given date. Sampling was conducted during April and May 2012 until the desired sample size was achieved.

Based on programme data, it was anticipated that there were twice as many PWID using the NSP in the Yamuna Bazar location in comparison to the other two locations. The selection of sampling events for the sampling calendar was weighted with probability proportionate to size, so that approximately half the sampling events would be drawn from the Yamuna Bazar location and the remaining half would be evenly split between Jehangipuri and Nabi Karim.

The data collection team consisted of one field supervisor, one enumerator and four interviewers who were responsible for recruiting and interviewing participants. During sampling events, the enumerator would use a “counter” to count every male who used the NSP to obtain new needles/syringes. Interviewers were directed by the enumerator to approach potential participants immediately after they had obtained new needles / syringes from the NSP. Not all people using the NSP during a sampling event could be approached to participate given there was a limited number of interviewers. Additionally, there would be more people attending the NSPs during some sampling events than others, thus creating a scenario of unequal probability of selection for participants across sampling events. The enumerator counted all people actually interviewed during each sampling event in addition to counting those who had used the NSP to enable the construction of selection probability weights as discussed in the data analysis section below. Additionally, the enumerator collected data on the number of people who were approached yet declined to participate and the number of participants who terminated the interview early.

### Data collection and measurement

The questionnaire was interviewer-administered to maximize the quality of the data collected. The questionnaire had five domains: 1) background information including socio-demographics, 2) drug use practices, 3) sexual behaviour, 4) quality of life, and 5) symptoms of depression, anxiety and suicidal ideation. It was anticipated that many of the participants would have never received any schooling. As a result, the questionnaire was kept as brief and simple as possible and it took approximately 30 minutes to complete. The questionnaire was translated into Hindi, back-translated into English and then piloted in the field to ensure equivalence and appropriateness of the questions and scales. Local research assistants were used for the data collection phase, all of whom had previously been involved in administering questionnaires with PWID. The research assistants were trained in using the questionnaire and ethical research conduct and were closely supervised by an experienced local research officer.

#### Demographics, injecting and sexual practices

Questions regarding demographics, drug use and sexual behaviours (predominantly condom use with various partners) were adapted from previous research [39] among injecting drug users in New Delhi and from a sub-set of questions adapted from the Integrated Biological and Behavioural Assessment (IBBA) survey previously undertaken among injecting drug users in three states of India, in both 2006 and 2009 [40].

#### Quality of life

Four generic questions relating to quality of life were asked, covering four different aspects of quality of life. The questions were adapted from themes contained within the WHOQoL scale [41] and the IDUQoL scale [42,43], neither of which proved feasible to use in this study during pilot testing due to the length of these instruments and challenges in translating concepts. The adapted questions were pertaining to: 1) hope for the future, 2) feeling safe in daily life (i.e. from crime and violence), 3) perception of health, and 4) relationship with family. Additional socio-economic variables that are also indicators of quality of life included: literacy, level of education, income, source of income, housing, history of imprisonment, experiences of being beaten and inclusion in family events.

#### Symptoms of depression, anxiety and suicidal ideation

Symptoms of depression were measured using the Patient Health Questionnaire (PHQ-9), a nine-item screening tool based on criteria for depressive disorders in the Diagnostic and Statistical Manual of Mental Disorders (DSM-IV) [44,45]. The PHQ-9 has well established criterion, construct and external validity [46,47]. The PHQ-9 asks participants whether they have been bothered with nine symptoms in the past two weeks, with response options on a four-category Likert scale; not at all (0), several days (1), more than half the days (2), nearly every day (3). The PHQ-9 is relatively short to administer with scores ranging from 0 to 27. Scores of 5, 10, 15 and 20 represent thresholds marking the lower limits of mild, moderate, moderately severe and severe depression.

The conventional cut-off is ≥10, yielding a sensitivity of 88% and a specificity of 88% for major depression [45]. The PHQ has previously been translated into Hindi, validated for use among South Asian populations and used in previous studies measuring the prevalence of depressive symptoms among various communities in India [48-52]. It has been recommended as a measure for symptoms of depression among PWID in NSP settings [53].

Symptoms of anxiety were measured using the Generalised Anxiety Disorder (GAD-2) scale, a two-item ultra-brief screening tool that follows the same format and response options as the PHQ-9 [54]. Scores on the GAD-2 range from 0 to 7 with a score of ≥3 representing the optimum cut-point for screening for anxiety disorders. The GAD-2 has high sensitivity (86%) and specificity (83%) for detecting generalised anxiety disorder and high specificity for panic disorder (81%), social anxiety disorder (81%) and post-traumatic stress disorder (81%) [54]. The GAD-2 has previously been translated into Hindi.

Suicidal ideation was captured using three questions adapted from the Suicide Behaviours Questionnaire (SBQ) [55]. Questions were asked pertaining to: 1) thoughts of suicide during the previous 12 months, 2) the frequency of such thoughts, and 3) whether or not a plan to act on these thoughts had been made. Additionally, participants were asked whether they had attempted suicide during the past 12 months.

### Sample size

With an assumed prevalence of psychological distress of 50% (the most conservative assumption for the sample size calculation) and a 95% confidence interval with a 5.5% margin of error, it was calculated that a sample size of 312 PWID would be required. TLS sampling is similar to cluster sampling, as randomly selected ‘blocks of time and space’ are potentially clusters of homogeneity. The required sample size was increased to 420 after adjusting for a design effect of 1.25.

### Data analysis

Data were entered into SPSS version 20 and cross-checking was undertaken among 10% of questionnaires to ensure there were no systematic errors when entering the data. Preliminary tables were generated for each variable to check for invalid entries. Data were analysed using Stata version 10. Survey commands in Stata were used to account for the TLS sampling design, specifically the effect of clustering within sampling events and the probability that a person was sampled [36,56]. Participants recruited at each sampling event (combination of time and location) were treated as a cluster and standard errors for variables were adjusted for intra-cluster correlation. Sampling events at different times but at the same location were treated as separate clusters. The number of attendees at a sampling location during each sampling event (i.e. enumeration count) was used as the basis upon which to construct selection probability weights. In brief, the first step was to calculate the proportion that each event represented of the total enumeration count for the entire study. The second step was to calculate the proportion of the total number of interviews that each event represented for the entire study. Finally, the result from step 1 was divided by the result from step 2 to generate a weight that represents the ratio of the number of persons enrolled into the study relative to the number of persons available to sample from at each sampling event. Thus, more weight was given to participants recruited at highly-attended sampling events.

Descriptive analysis produced adjusted proportions and means with their respective 95% confidence intervals (C.I.). The range and median were used to provide important information as to the distribution of the continuous depressive and anxiety symptom measures. Because these statistics could not be generated using survey commands in Stata, due to the limited options available in these commands, they were generated without adjustment for the sampling design.

## Results

### Recruitment

There were 420 PWID recruited into the study: 220 (52%) from Yamuna Bazar, 100 (24%) from Nabi Karim and 100 (24%) from Jehangipuri. During recruitment there were 173 PWID who were approached but declined to participate; if we take a conservative assumption that all 173 met the inclusion criteria, this would indicate a response rate of 71% (420 ÷ (420 + 173)). There were no occasions where a participant terminated the interview before it was completed.

### Demographics and substance use

Table 1 displays the demographic characteristics and substance use practices of PWID in this study setting. The mean age was 36.7 (95% C.I. 35.5-37.9). Only one in nine (11%) was below the age of 25 and one-quarter were aged 45 or older (26%). Many PWID were born outside of New Delhi, with just over one-third (38%) being born there. Just over half (53%) had never been married and nearly one-quarter were either divorced / separated (16%) or widowed (7%); more than half (60%) had no children.

**Table 1:** Demographics and substance use (n = 420)

**Category**	**% (95 % C.I.)**
**Age**	
*18*–*24*	11.3 (7.1-15.6)
*25*–*34*	34.4 (28.2-40.6)
*35*–*44*	28.7 (23.7-33.6)
*45*+	25.6 (19.8-31.4)
**Place of birth**	
*New Delhi*	38.3 (26.7-50.0)
*Uttar Pradesh*	27.9 (22.2-33.6)
*Other*	33.7 (25.2-42.3)
**Mother tongue**	
*Hindi*	74.4 (67.5-81.3)
*Punjabi*	6.5 (3.6-9.5)
*Bengali*	4.6 (1.95-7.3)
*Nepali*	4.3 (2.1-6.5)
*Other*	10.2 (6.0-14.4)
**Marital status**	
*Never married*	53.3 (46.4-60.1)
*Currently married*	24.6 (17.8-31.4)
*Divorced* / *separated*	15.5 (12.0-19.0)
*Widowed*	6.6 (3.0-10.2)
**Number of children**	
*None*	60.3 (53.7-66.9)
*1*–*2 children*	26.4 (22.0-30.9)
*3*–*4 children*	10.4 (6.3-14.5)
*5*+	2.9 (0.6-5.2)
**Current tobacco use**	
[smoking, chewing or in paan]	
*Daily*	93.2 (90.1-96.2)
*Less than daily*	4.9 (2.3-7.6)
*Not at all*	1.9 (0.5-3.3)
**Alcohol use** – **past month**	
*Have never consumed alcohol*	21.1 (14.4-27.7)
*Not used in past month*	43.4 (36.1-50.7)
*Less than once a week*	9.5 (6.9-12.1)
*At least once a week*	19.1 (14.4-23.7)
*Everyday*	7.0 (3.3-10.7)
**Age at first injection**	
≤*13*	34.1 (29.1-39.0)
*14*–*16*	26.2 (21.6-30.8)
*17*–*19*	18.3 (13.7-22.9)
*20*+	21.5 (15.9-27.1)
**Drugs used in the last 3 months **^a^	
*Pheniramine maleate*	99.0 (98.1-100.0)
*Buprenorphine*	97.4 (95.4-99.3)
*Heroin*	76.6 (69.9-83.3)
*Marijuana*	73.0 (63.4-82.7)
*Diazepam*	53.0 (44.8-61.1)
*Promethazine*	42.3 (35.6-48.9)
*Spasmoproxyvon*	26.5 (19.1-33.9)
*Pentazocine* / *morphine*	25.1 (19.6-30.6)
*Other*	22.2 (17.1-27.4)
**Main drug injected in last 3 months **^b^	
*Buprenorphine base*	76.9 (69.7-84.1)
*Heroin base*	18.0 (11.6-24.3)
*Other*	5.2 (1.7-8.6)
**Number of times injected during past week**	
≤*7*	14.4 (10.4-18.3)
*8*–*14*	28.7 (23.0-34.3)
*15*–*21*	36.4 (30.9-42.0)
*22*+	20.5 (15.5-25.5)

^a^ Buprenorphine, heroin, morphine, pentazocine (common brand name is Fortwin) and Spasmoproxyvon are synthetic opioid analgesics. Spasmoproxyvon is the brand name for a compound drug containing dextropropoxyphene, dicyclomine hydrochloride and paracetamol. Pheniramine maleate (common brand name is Avil) and promethazine (common brand name is Phenergan) are antihistamines.

^b^ Both buprenorphine and heroin are commonly injected in a cocktail combination with a liquid antihistamine and often diazepam as well.

Tobacco was consumed daily for the majority of these PWID (93%). Few (7%) consumed alcohol daily. The mean age of first injection was 15.8 (95% C.I. 15.3-16.3) and one-third (34%) had first injected at the age of 13 or younger. The mean length of injecting career was 20.9 years (95% C.I. 19.8-22.0). The most commonly used opioids in the last three months were buprenorphine (97%), heroin (77%), Spasmoproxyvon (27%) and pentazocine / morphine (25%). Buprenorphine, Spasmoproxyvon and pentazocine / morphine are synthetic opioid analgesics commonly found in chemist shops. Spasmoproxyvon is the brand name for a compound drug containing dextropropoxyphene, dicyclomine hydrochloride and paracetamol. Other commonly used drugs were marijuana (73%), diazepam (53%) and antihistamines (pheniramine maleate / Avil 99%, promethazine / Phenergan 42%). The main drug injected in the last three months was typically buprenorphine (77%) or heroin (18%), both of which were commonly injected in a cocktail with a liquid antihistamine and often diazepam as well. Few (14%) PWID were injecting only once a day or less.

### Quality of life and defining socio-economic characteristics

Table 2 presents the quality of life and socio-economic characteristics, showing an extremely impoverished and marginalised community of PWID. Just one-fifth of PWID felt very hopeful for their future (21%) or felt very safe in their daily life from violence, crime and harassment (18%); over one-quarter reported feeling not at all safe (28%). Few rated their personal health as either very good (1%) or good (11%). Similarly, few rated their relationship to family as either very good (3%) or good (14%) and the majority reported that they either rarely (27%) or never (45%) attend family events.

**Table 2:** Quality of life and socio-economic indicators (n = 420)

**Category**	**% (95% C.I.)**
**Feel hopeful for your future**	
*Very hopeful*	21.1 (15.5-26.7)
*Somewhat hopeful*	60.0 (55.5-64.4)
*Not at all hopeful*	19.0 (14.4-23.5)
**Feel safe in your daily life**	
[safe from violence, crime and harassment]	
*Very safe*	17.7 (13.0-22.4)
*Somewhat safe*	54.2 (47.6-60.7)
*Not at all safe*	28.2 (21.8-34.6)
**Rating of personal health**	
*Very good*	0.7 (0.0-1.4)
*Good*	10.9 (7.3-14.6)
*Average*	45.9 (40.7-51.2)
*Poor*	26.8 (22.1-31.4)
*Very poor*	15.6 (10.3-20.9)
**Relationship with family**	
*Very good*	3.0 (1.1-5.0)
*Good*	13.5 (9.8-17.2)
*Average*	43.0 (37.7-48.4)
*Poor*	21.3 (15.5-27.1)
*Very poor*	19.2 (13.0-25.3)
**Included in family events**	
*Always*	16.4 (9.6-23.2)
*Some or most of the time*	11.1 (7.8-14.3)
*Rarely*	27.1 (22.9-31.3)
*Never*	45.4 (38.4-52.5)
**Literacy**	
*Literate*	37.8 (56.7-67.6)
*Illiterate*	62.2 (56.7-67.6)
**Years of primary and secondary education completed**	
*Never attended school*	38.0 (32.7-43.3)
*Completed between 1*–*4 years*	28.0 (22.9-33.2)
*Completed between 5*–*11 years*	32.1 (27.4-36.8)
*Completed 12 years*	1.8 (0.6-3.1)
**Daily income** (**rupees**)^a^	
≤*100 rupees*	21.9 (16.7-27.1)
*101*–*150 rupees*	26.8 (21.7-32.0)
*151*–*200 rupees*	24.3 (19.8-28.8)
*201*–*250 rupees*	11.9 (7.8-16.0)
*251*+ *rupees*	15.1 (10.7-19.6)
**Main source of income**	
*Skilled manual work*	10.9 (6.3-15.5)
*Unskilled manual work*	18.7 (11.7-25.8)
*Scavenging* (*e*.*g*. *rag picking*)	47.8 (37.5-58.2)
*Trading* / *vending*	1.8 (0.5-3.2)
*Crime* (*e*.*g*. *pick pocketing*, *selling drugs*, *etc*.)	5.7 (2.4-9.0)
*Begging*	1.7 (0.1-3.3)
*No source of income*	11.1 (7.1-15.1)
*Other*	2.2 (0.1-3.9)
**Place where slept most in past 3 months**	
*Homeless*	68.8 (57.4-80.3)
*House* / *flat of relative or friend*	16.0 (8.6-23.4)
*Own house or flat*	5.9 (1.6-10.2)
*Rented room* (*hotel or rooming house*)	5.7 (3.1-8.2)
*Other temporary housing*	3.6 (1.8-5.3)
**Ever been imprisoned**	
*No*	30.4 (27.2-33.5)
*Yes*	69.6 (66.5-72.8)
**Number of times beaten up in the past 6 months**	
*0*	50.7 (44.5-57.0)
1	15.7 (10.6-20.9)
*2*	13.2 (9.5-16.9)
*3*	9.3 (6.4-12.1)
*4*+	11.0 (8.4-13.5)

^a^ 1.00 USD = 54.25 rupees [as of 9th May 2013].

The level of illiteracy was very high (62%) and just 2% had completed 12 years of primary and secondary education. On average, PWID in this community live off a mean of 193.5 rupees per day (95% C.I. 181.1-206.0), roughly $US3.50 and approximately half had a daily income of ≤100 rupees (22%) or 101–150 rupees (27%). Scavenging / rag picking was the main form of income for 48% of PWID and just under one-third did either unskilled manual work (19%) or skilled manual work (11%). A small group of PWID primarily generated their income from either crime (6%) or begging (2%).

There was a very high proportion of PWID who were homeless (69%) and several others mostly slept in insecure housing including rented rooms (6%) and other temporary housing (4%). Seventy percent had a history of imprisonment. Experiences of physical violence were common: 33% had been beaten up at least twice during the preceding six months and 11% had been beaten up four or more times.

### Depression, anxiety and suicidal ideation

Table 3 presents the prevalence of recent symptoms of depression and anxiety and the 12-month prevalence of suicidal ideation. The PHQ-9 scale had satisfactory internal consistency (Cronbach’s alpha coefficient = 0.73). The mean PHQ-9 score was 14.7 out of a total possible score of 27, with a range of 1 to 25 and a median of 15. The prevalence of moderate or worse symptoms of depression (cut-off ≥10) was 84% and the prevalence of moderately severe to severe symptoms of depression (cut-off ≥15) was 54%.

**Table 3:** Prevalence of recent symptoms of depression and anxiety, and 12-month prevalence of suicidal ideation (n = 420)

**Category**	
**Depression and anxiety scales** - **averages**	**Mean (95% C.I.)**
PHQ-9 score: average	14.66 (13.68-5.65)
GAD-2 score: average	3.44 (3.20-3.68)
**Depression and anxiety scales** – **cut points**	**% (95% C.I.)**
PHQ-9 score: ≥10	84.4 (79.6-89.2)
PHQ-9 score: ≥15	54.2 (45.4-63.0)
PHQ-9 score: ≥20	17.4 (10.7-24.1)
GAD-2 score: ≥3	70.5 (64.3-76.8)
**Suicidal ideation**	**% (95% C.I.)**
Have you thought about killing yourself in the past 12 months?	
*No*	46.9 (41.9-52.0)
*Yes*	53.1 (48.0-58.1)
How often have you thought about killing yourself in the past 12 months?	
*Never*	46.9 (41.9-52.0)
*Rarely* (*1 time*)	8.8 (5.4-12.2)
*Sometimes* (*2 times*)	22.1 (18.1-26.1)
*Often* (*3*–*4 times*)	15.1 (11.0-19.3)
*Very often* (*5 or more times*)	7.0 (4.2-9.9)
Have you ever made a plan to kill yourself in the past 12 months?	
*No*	66.3 (61.4-71.2)
*Yes*	33.7 (28.8-38.6)
Have you ever attempted to kill yourself in the past 12 months?	
*No*	63.8 (58.6-68.9)
*Yes*	36.3 (31.1-41.4)

Note: PHQ-9 scores of ≥10, ≥15 and ≥20 represent cut points for moderate, moderately severe and severe depression symptomology. A GAD-2 score of ≥3 represents the optimum cut point when screening for anxiety.

Among the nine items on the PHQ-9, the highest average score was for the item “feeling bad about yourself – or that you are a failure or have let yourself or your family down”; the mean was 2.2 (95% C.I. 2.1-2.3). The proportion of participants who received the maximum score of 3 (i.e. had this symptom nearly every day in the last two weeks) for this item was 51% (95% C.I. 44%-58%).

The mean GAD-2 score was 3.4 out of a total possible score of 6, with a range of 0 to 6 and a median of 4. Seventy-one percent qualified as having anxiety symptomatology (cut-off score of ≥3). There was a small group (10%, 95% C.I. 6%-14%) of PWID who displayed no recent (i.e. past two weeks) symptoms of either depression or anxiety.

Suicidal ideation during the past 12 months was common. Fifty-three percent thought about killing themselves and almost one-quarter either often (15%) or very often (7%) thought about killing themselves. A substantial proportion reported to have actually attempted to kill themselves (36%) in the past 12 months.

## Discussion

The findings from this study profile an impoverished, vulnerable and isolated population of men who inject drugs and whose lives are shaped by a significant level of psychosocial distress. The average age of participants was in the mid-30s with few aged below 25 and most had commenced their injecting careers during adolescence, indicative of a population of PWID who belong to the more chronic end of the addiction spectrum. The prevalence of symptoms of depression and anxiety was very high and suicidal thoughts and acts were disconcertingly common.

### Low quality of life and socio-economic status

More than half the participants were born outside of Delhi, suggesting many of these men were part of the phenomenon of large-scale migration into the city whether as adults or children. Migration into Delhi has fuelled a population growth rate of between 4-5% per year between 1951 and 2001 [57], making Delhi the home to 16.7 million people [58] and a megacity that ranks among the largest on the planet.

In our study, the quality of life and socio-economic indicators reveal a socially excluded population of PWID in Delhi who have minimal education and are often homeless, leaving them vulnerable to physical violence, poverty, poor health and imprisonment. These men are clearly excluded from the rapid social and economic progress around them.

The social exclusion extends to family relationships, the core of the social fabric in India [59], with many among this population never (45%) or rarely (27%) attending family events. Aside from the potential for breakdown in family relationships in connection with migration, there are clearly other explanations connected to drug addiction and family dynamics. The heightened level of stigma, shame and alienation associated with injecting drug use in a conservative Indian society would threaten the integrity of a family and deepen the fracturing of relationships [60]. To the best of our knowledge there is minimal research in India, if any, examining drug users’ perspectives regarding their relationships with family and the potential for meaningful engagement of family in drug treatment linked to harm reduction. Given the pivotal role of family in Indian society, there would be value in a more thorough examination of both the drug users’ and family-members’ perspectives on drug dependence and family relationships.

### A high prevalence of common mental health symptoms

There was a high prevalence of symptoms of depression and anxiety among this community-based population of men who inject drugs. In our study, 84% (PHQ-9 ≥10) had symptoms of depression, 54% (PHQ-9 ≥15) had moderately severe or severe symptoms of depression and 71% (GAD-2 ≥3) had symptoms of anxiety. The most common symptom of depression was feelings of worthlessness and self-blame which is not very surprising given the highly stigmatised nature of injecting drug use, the disconnection with family and the deprived socio-economic circumstances for many among this population, but is in contrast to the findings of a population-based survey of depression in Chennai that also used the PHQ and reported depressed mood as the most common symptom [51].

These results are somewhat comparable to studies conducted in high-income countries among PWIDs using self-report symptoms scales, despite variation in study settings and the particular scale used. Lemstra et al. [22] used the CES-D (cut-offs ≥16, ≥23) among PWID in the Saskatoon Health Region of Canada and found a point prevalence of depressive symptomatology of 81%, with 58% having more severe depressive symptoms. Perdue et al. [23] also used the CES-D (cut-off ≥23) among PWID in Seattle (USA) and similarly found that 47% had more severe depressive symptoms. Brienza et al. [24] used the Structured Clinical Interview for DSM-III-R (SCID) and found a point prevalence of major depression over the last 6 months of 54% among PWID in Rhode Island (USA) attending needle syringe exchange programmes and 42% among PWID attending methadone maintenance treatment. Reyes et al. [25] used the Beck Anxiety Inventory (moderate levels 16–25, severe levels ≥26) among PWID in Puerto Rico; over half of participants were found to have either moderate (19%) or severe (37%) levels of anxiety.

Similarly high prevalence rates have been found in other studies conducted in the Asian region. Gu et al. [28] used the Depression Anxiety and Stress Scale (DASS) among a snowball sample of females who inject drugs in Dazhou city (Sichuan Province, China) and found a high percentage of participants reported depressive symptoms (75% to 92%). Liao et al. [29] used the Zung Self-Rating Anxiety Scale (SAS) and the Zung Self-Rating Depression Scale (SDS) among a sample of heroin-dependent patients in a treatment research clinic in Changsha city (Hunan Province, China) and found a high prevalence of both high anxiety (53%) and depression (43%) symptoms.

Our study revealed a disconcertingly high prevalence of suicidal ideation. We found that 53% had thought about killing themselves in the past 12 months and 34% had made a plan to kill themselves. This result is the same as that found by Sarin et al. [31] who measured suicidal ideation among PWID in Delhi whilst conducting a study focused on human rights abuses: 57% had thought of taking their own life in the past six months and 42% reported having made a specific plan about how they would take their own life. High rates of suicidal ideation have been found among PWID elsewhere: Havens et al. [26] surveyed a sample of PWID in Maryland (USA) and found that 27% had thought about killing themselves in the past 6 months.

Our study also found that many (36%) men who inject drugs in Delhi had moved beyond thinking about killing themselves to actually attempting to kill themselves in the past 12 months. Suicide is a real option for these men, with at least one means readily available in the form of a drug overdose. Chen et al. [30] also found that suicide attempts were common among heroin users in Taiwan; they surveyed entrants to methadone maintenance treatment and found that 10.9% had attempted suicide in the previous month.

### Implications for programmes seeking to integrate a focus on mental health

The high prevalence of psychological distress found in this study has implications for programmes seeking to engage, treat and rehabilitate PWID in India. Two broad approaches appear to be worth further consideration, although both face substantial challenges. One approach to integrating mental health into services for PWID would be to adopt a conventional psychiatric biomedical model based around the use of screening tools and referral mechanisms complimented by the development of mental health literacy [61,62] and mental health first aid initiatives [63,64] for programmes providing services to PWID. In an ideal world the development of locally appropriate and easily administered and interpreted mental health screening tools could provide a quick method for programmes to identify PWID with severe symptoms of common mental health problems. Screening tools could theoretically be complemented by the development of pathways of referral for mental health assessment at the primary health care level or, where appropriate, through psychiatric institutions.

However, there is a question mark over whether conventional screening and treatment approaches would be effective for people who live in such adverse circumstances. Separating human distress from a diagnosis of depression or anxiety is difficult [65]. Furthermore, given that the unmet need for mental health care in India is very high [4], it is unlikely that this disenfranchised population group will be able to reliably access psychiatric or psychological treatment when needed without strong advocacy and positive relationships with treatment providers. The sheer number of new referrals that may be generated from the regular use of screening tools among this population group might overwhelm already stretched mental health services. Additionally, there may be obstacles to persuading PWID to attend assessments and treatments and there may be financial barriers related to the costs of assessment and treatment.

Whilst severe depression and anxiety may require psychiatric treatment, those with milder to moderate symptoms could be better served by the adoption of a broader public health approach that targets the population rather than the individual [66]. The concept of mental health promotion is a possible alternative to the conventional psychiatric biomedical model. A mental health promotion approach would involve addressing the social determinants of mental health and well-being, those being social inclusion, freedom from discrimination and violence and economic participation [67]. Structural interventions seeking to improve access to social/group activities and addressing unemployment and housing insecurity could be critical strategies for addressing mental health among this population of men who experience multiple levels of deprivation.

### Limitations

The use of NSPs as the primary site of participant recruitment may have created a sampling bias. Those PWID who participated in this study are more likely to be known to services and may represent the more chronic end of the addiction spectrum. The method of participant recruitment may have missed out on casual users and those who were newer to injecting drug use. Additionally, those who declined to participate may differ from those who did participate and we are unable to assess the degree to which our participants were representative of the broader NSP client group; however, a response rate of 71% is acceptable and suggests the sample is satisfactorily representative.

Symptoms of depression and anxiety were measured using self-report screening tools rather than a formal psychiatric diagnosis. The selection of the optimal tool for evaluating comorbid psychiatric conditions in drug using populations remains controversial. In our study, it is not possible to differentiate between psychiatric symptoms that are independent of substance use and those that were a consequence of it. Furthermore, the high level of psychological distress among our study participants is not surprising given their significant socioeconomic stress, disconnection from family, and the stigma connected to illicit drug addiction. Jacobs [66] argues that symptom checklists, as used in this study, take little regard of context, stress and coping, and that separating human distress from depression is difficult. The proportion of the participants who would reach a formal diagnosis is unknown, and many may be experiencing psychological distress related to their socially adverse circumstances and may not reach diagnostic criteria. Finally, we note that the use of a comparison group in our study design and double-entry at the point of data entry would have strengthened the methods.

## Conclusions

Our study into men who inject drugs in Delhi revealed an impoverished, vulnerable and isolated population whose lives are shaped by a significant level of psychosocial distress. The prevalence of depressive and anxiety symptoms among this population of men who inject drugs in Delhi was very high and suicidal thoughts and acts were disconcertingly common. Given the above and the high level of unmet need for mental health care in India, further research and discussion is required to inform the most appropriate public health response to the mental health and wellbeing needs of this population group.

## Competing interests

The authors declare that they have no competing interests.

## Authors’ contributions

GA and MK designed the study and conceptualized the analysis in this paper. GA developed the sampling strategy and supervised the implementation of data collection in collaboration with LS, SS and AM. AM entered and cleaned the data under the supervision of GA. GA undertook the data analysis and wrote the first draft of this paper. LS and SS provided links to the injecting drug user community and data collection sites, and contributed fundamentally important contextual knowledge that informed the development and implementation of the study. All authors read and contributed to the final manuscript.

## Pre-publication history

The pre-publication history for this paper can be accessed here:

http://www.biomedcentral.com/1471-244X/13/151/prepub
